# Enhancing Surgical Operative Note Standards at Doka Hospital, Sudan: A Clinical Audit

**DOI:** 10.7759/cureus.72441

**Published:** 2024-10-26

**Authors:** Hussam Mohamed Hassan Ibrahim, Ali Abdellateef Abdelrahman Zeineldeen, Mahmood Abdalmonem Mohamed Mohamed Alameen, Izzut Awad Ahmed Abdullah, Heyam Abd Elsalam Mahmoud Saeed, Mohamed Fawzi Osman Khalid, Hadeel Abd Alsalam Mahmoud Saeed, Mohammed Hassan Morsal Maali, Ali Suliman Ali Bitay, Aymen Abdelhakim Ahmed Mohammed

**Affiliations:** 1 General Surgery, Doka Hospital, Elgadarif, SDN

**Keywords:** audit cycle, clinical documentation audit, operative note, quality improvement, surgical documentation

## Abstract

Background

Operating notes are crucial in surgical practice for post-operative care, future medical reference, and legal needs. They provide vital details about the procedure, guaranteeing patient safety and improving communication among medical staff. These notes include patient health status, surgical method, operation results, problems, and post-surgery plans. Established protocols for recording surgical operative notes are essential for medical education, research, and quality improvement efforts. The audit aimed to evaluate and enhance Doka Hospital's surgical operation notes, focusing on their quality, completeness, and potential areas for development. The audit aimed to apply necessary adjustments to raise standards and ensure high standards in medical treatment.

Material and methods

This retrospective-prospective study analyzed surgical postoperative notes from Doka Hospital's archives, following the implementation of a new format based on guidelines set by the Royal College of Surgeons of England. Staff training sessions were conducted to facilitate adoption. The audit population included surgical operative notes from the departments of general surgery and obstetrics and gynecology. The study collected 200 surgical operative notes, with 100 notes in the first cycle and 100 notes in the second cycle. Data was collected from the General Surgery and Obstetrics and Gynaecology departments using a predefined checklist, followed by analysis using the Microsoft Excel Sheets program (Microsoft Corporation, Redmond, USA). Ethical clearance was obtained from the ethical committee of Doka Hospital. The audit population consisted of 71 beds in a busy rural hospital with a majority of cases involving obstetrics and one-day surgical operations.

Results

Doka Hospital's surgical operative note review showed a significant improvement in documentation quality following a revised structure based on the Royal College of Surgeons of England's recommendations. Compliance with the criteria improved from 50.5% in the first cycle to 82.5% in the second cycle. Notable progress was made in date and time documentation, with a 96% increase in the second cycle. The proportion of anaesthetists' names also rose from 60% to 90%. The accuracy of recording surgical operation details increased from 87% to 94%. Documenting operational results, issues, and tissue removal also improved. Factors like the identity of the prosthesis or materials used remained unchanged in both cycles.

Conclusion

The introduction of a uniform style for recording surgical operational notes, together with focused training sessions, greatly improved the quality of surgical documentation at Doka Hospital.

## Introduction

Operative notes are critical documents that record the details of surgical procedures, playing a significant role in ensuring patient safety and effective communication among healthcare providers. According to the World Health Organization (WHO), accurate documentation of surgical procedures is vital for improving surgical outcomes and minimizing complications [1]. High-quality operative notes are essential for clinical governance, as they provide a legal record of the procedure and inform postoperative care. According to the Royal College of Surgeons of England (RCS), operative notes should include key details such as the patient's demographics, the procedure performed, findings, and postoperative care instructions [2].

A crucial medical document of exceptional clinical, medico-legal, and scholarly significance is an operating note. To standardize operating notes, the Royal College of Surgeons (RCS) has established standards. This closed-loop audit evaluated how well our local hospital's operative notes adhered to the RCS guidelines in order to find shortcomings and enhance procedures [3].

Operation note documentation is essential for patient safety since it summarizes the most important results and minute details of a surgical approach. The postoperative patient care might be adversely affected by inadequate operation note documenting. Examining manual operation note documenting practices was the goal of this investigation [4].

Auditing surgical operative notes is crucial for ensuring accurate, consistent, and complete documentation in surgical practice. Well-maintained notes are essential for patient safety, continuity of care, legal accountability, and effective communication among healthcare professionals. Auditing helps identify gaps in documentation, such as missing critical details, unclear descriptions, or inconsistencies in procedures. By regularly auditing these notes, healthcare teams can standardize best practices, improve compliance with guidelines, reduce errors, and ultimately enhance the quality of patient care. It also supports a culture of continuous improvement and accountability in surgical departments [5,6].

## Materials and methods

This retrospective-prospective study was conducted in the General Surgery and Obstetrics and Gynecology Departments of Doka Hospital, Elgadarif State, Sudan, a busy rural hospital with a capacity of 71 beds. The Institutional Review Board at Doka Hospital provided ethical approval for this investigation (#2023-DA01). Our study's objectives were to evaluate surgical operational notes' quality in light of the Royal College of Surgeons' (RCS) requirements (Table 1), spot documentation gaps, and make organized interventions to enhance the proforma format.

**Table 1:** Checklist for parameters of operative notes according to Royal College of Surgeons recommendations. Source: [2] DVT: deep vein thrombosis

Parameters Checklist
Date and time
Type of operation
Name of operator and assistant
Name of anaesthetist
Operative Procedure carried out
Incision type
Operative diagnosis
Operative finding
Problems/Complications
Any extra procedures and why it was performed
Details of tissue removed, added or altered
Identification of Prostheses/Material used
Details of closure technique
Anticipated blood loss
Prophylaxis antibiotics
DVT Prophylaxis
Details of postoperative care instructions
Surgeon’s Signature

Aim and objectives

This clinical audit aims to evaluate and enhance the quality of operative notes within surgical departments, identify deficiencies, and propose actionable recommendations based on established best practices. By addressing the gaps in documentation, the study seeks to enhance surgical practices and improve patient safety.

Audit area/population

The audit was conducted at Doka Hospital, which primarily handles obstetric cases and one-day surgical operations. Surgical operative notes from the General Surgery and Obstetrics and Gynecology Departments were reviewed during two separate audit periods: the first cycle was conducted retrospectively over three days in October 2023, while the second cycle was conducted prospectively over the month of November 2023. The focus was on assessing adherence to RCS standards in documenting postoperative details.

Sample size and sampling technique

A total of 200 surgical operative notes were analyzed for this study, with 100 notes reviewed retrospectively in the first cycle over three days in October 2023, and another 100 notes reviewed prospectively in the second cycle over the month of November 2023. The sampling was done using a simple random sampling technique to ensure that the notes selected for both cycles represented the broader population of cases handled in these departments. The samples included both major and minor cases from General Surgery and Obstetrics and Gynecology, without distinction between the two departments.

Data collection method and analysis

Data collection was carried out by trained medical professionals who meticulously reviewed the surgical notes using a predefined checklist developed in line with the Royal College of Surgeons of England's recommendations. The checklist assessed 18 specific parameters, focusing on the completeness and accuracy of the documentation. The first cycle involved a retrospective review of notes from 100 randomly selected cases in October 2023, and the second cycle was a prospective review conducted after implementing a revised proforma and conducting staff training sessions to ensure proper documentation practices. The data was analyzed using Microsoft Excel 2016 (Version 16.0; Microsoft Corporation, Redmond, Washington, United States) to evaluate improvements in documentation between the two cycles.

Ethical clearance

Ethical clearance for this audit was obtained from the Institutional Review Board of Doka Hospital under IRB number 2023-DA01.

## Results

Following the revised structure outlined by the Royal College of Surgeons of England, the review of Doka Hospital's surgical operative notes showed a significant improvement in the quality of documentation between the first and second audit cycles. In the first cycle, compliance with the documentation criteria was 50.5%, which increased substantially to 82.5% in the second cycle. This review examined a total of 200 surgical notes, with 100 notes assessed in each cycle. The results highlighted considerable improvements in nearly all areas of surgical documentation, indicating the positive impact of the implemented interventions.

A closer look at specific criteria reveals the substantial progress made. The documentation of the date and time of operations, for example, increased from 55% in the first cycle to 96% in the second cycle, reflecting a 41% improvement. This criterion is particularly critical, as accurate timing is essential for tracking and coordinating patient care. Similarly, the documentation of the type of operation showed an impressive 92% improvement, rising from 0% in the first cycle to 92% in the second. This sharp rise signifies that the training interventions effectively targeted one of the most neglected areas of documentation.

The name of the anesthetist also saw a significant increase, improving from 60% to 90%, a 30% rise, indicating better acknowledgment of the full surgical team in the operative notes. Documentation of operative procedures carried out improved by 11%, from 83% to 94%, and the incision type documentation increased by 19%, from 70% to 89%. These improvements are crucial as they enhance clarity in surgical records, aiding in post-operative care and future reference for any subsequent procedures.

Further gains were noted in documenting operative findings, which rose by 21%, from 67% to 88%. This improvement ensures that important intraoperative details are recorded comprehensively. Similarly, documenting problems or complications during the surgery jumped from 41% in the first cycle to 82% in the second, reflecting a 41% improvement, which is critical for understanding intraoperative challenges and their management.

Some of the most dramatic improvements were seen in areas such as the details of tissue removed, added, or altered, which surged by 60% (from 25% to 85%), and the documentation of prophylactic antibiotics, which increased by 45% (from 55% to 100%). The recording of closure techniques saw a 52% increase, rising from 48% in the first cycle to a perfect 100% in the second cycle. These findings demonstrate enhanced attention to critical aspects of patient safety and surgical outcomes.

However, certain areas, such as the identification of prostheses or materials used, saw no change, remaining at 0% in both cycles. This lack of improvement suggests that this area was not sufficiently addressed by the interventions and may require more focused attention in future audits. Similarly, the documentation of postoperative care instructions slightly declined from 78% to 70%, indicating a need for further refinement in this aspect of surgical note-keeping.

Overall, the implementation of new documentation styles and training courses contributed to a significant enhancement in the depth, accuracy, and completeness of surgical operative notes at Doka Hospital. These improvements are likely to translate into better patient outcomes, as comprehensive and precise documentation is vital for effective postoperative care and long-term patient management. The new documentation style and training courses clearly improved the depth and accuracy of surgical operative notes as seen in (Table 2).

**Table 2:** Illustrating the documentation outcomes from both the first and second audit cycles along with the corresponding percentage improvement achieved between them. DVT: deep vein thrombosis

Criteria	1st cycle	2nd cycle	Improvement
Date and time	55 %	96 %	41%
Type of operation	0 %	92 %	92%
Name of operator and assistant	90 %	97 %	7%
Name of anaesthetist	60 %	90 %	30%
Operative Procedure carried out	83 %	94 %	11%
Incision type	70 %	89 %	19%
Operative diagnosis	96 %	99 %	3%
Operative finding	67 %	88 %	21%
Problems/Complications	41 %	82 %	41%
Any extra procedures and why it was performed	12 %	61 %	49%
Details of tissue removed, added or altered	25 %	85 %	60%
Identification of Prostheses/Material used	0	0	0
Details of closure technique	48 %	100 %	52%
Anticipated blood loss	34 %	57 %	23%
Prophylaxis antibiotics	55 %	100 %	45%
DVT Prophylaxis	26 %	85 %	59%
Details of postoperative care instructions	78 %	70 %	-8%
Surgeon Signature	69 %	100%	31%

## Discussion

The results of our study, conducted at Doka Hospital, demonstrate a significant improvement in the quality of surgical operative notes following the intervention based on the Royal College of Surgeons of England (RCS) guidelines. The improvement from 50.5% compliance in the first cycle to 82.5% in the second cycle underscores the effectiveness of the implemented measures, which included introducing a structured format and conducting training sessions. This mirrors the results seen in other studies where adherence to structured guidelines significantly improved documentation quality.

In a study by Burney et al., a similar intervention approach was adopted to improve the quality of orthopedic operative notes, where compliance improved from 40.7% in the first cycle to 64.8% in the second cycle. This increase, though notable, was smaller compared to our study, where the improvement was more pronounced. The greater improvement in our study could be attributed to the comprehensive nature of our intervention, which included both a revision of the format and focused training sessions. Furthermore, while Burney et al. reported improvements in 20 out of 22 parameters, compliance in documenting prosthesis details remained poor. In contrast, our study did not report any improvement in the documentation of prostheses or materials used, indicating a shared challenge across studies in ensuring adherence to this parameter [7].

El-Hakim et al. introduced an electronic system to improve operative note documentation, achieving significant improvements in legibility, which increased from 71% to 96%. Although our study did not directly address legibility, the implementation of a standardized format likely contributed to improved clarity in the documentation. Moreover, the shift from 55% to 96% in documenting the date and time of surgery in our study closely mirrors the improvements seen with the introduction of electronic notes in El-Hakim's study. Despite the advances made with electronic systems, our study demonstrates that substantial improvements can also be achieved through the use of structured templates and training, without the need for technological interventions [8].

Zahid et al. conducted a similar closed-loop audit and observed improvements in several key documentation aspects, including surgical complications, extra procedures, and post-operative care instructions. Our findings also showed notable improvements in these areas. For example, the documentation of problems or complications increased from 41% to 82% in our study, while Zahid et al. reported significant enhancements in recording complications. However, our study showed a drop in documenting post-operative care instructions, which decreased from 78% to 70%. This could be due to a possible focus shift toward improving intra-operative documentation, as post-operative care instructions were already well-documented in the first cycle. This suggests that future interventions may need to ensure that improvements are balanced across all aspects of documentation [9].

A study by Hassan et al. focused on improving documentation through the introduction of a structured proforma, reporting a significant increase in overall documentation completeness from 65.2% to 95.2%. The use of proformas and training, as demonstrated in both our study and that of Hassan et al., has proven to be an effective approach for improving compliance with documentation standards. Our results align closely with the findings of Hassan et al., particularly in terms of the dramatic improvements in areas such as operative findings and closure technique. However, similar to their study, we noted persistent challenges with documenting prostheses, indicating this may be a widespread issue in surgical documentation [10].

Okoli and Adetula reported high compliance (90%-100%) with several parameters in their initial audit, including the documentation of the operating surgeon, diagnosis, and signature. Similarly, in our study, high compliance was achieved in documenting the names of the operators and assistants, with an increase from 90% in the first cycle to 97% in the second. However, both studies noted poor compliance in documenting prostheses, suggesting that this parameter is consistently under-addressed across different settings. Additionally, Okoli and Adetula did not observe significant improvements in areas that had already been poorly documented in their first cycle. This is somewhat at odds with our findings, where substantial improvements were made in previously under-documented areas, such as incision type (from 70% to 89%) and details of tissue removed, added, or altered (from 25% to 85%) [11].

Overall the implementation of a structured format and targeted training led to a significant improvement in the quality of surgical operative notes at Doka Hospital, increasing overall compliance with RCS guidelines from 50.5% in the first cycle to 82.5% in the second. However, challenges remain in specific documentation areas, such as prosthesis details, which showed no improvement.

Certain parameters, such as operative diagnosis (96% to 99%), problems or complications (41% to 82%), and details of closure technique (48% to 100%), exhibited improvements, reflecting a successful intervention strategy. 

However, not all parameters demonstrated improvement. Notably, the documentation of prostheses or materials used remained stagnant at 0% in both cycles. This lack of change could be attributed to a general lack of care and attention from doctors in this specific area, as well as insufficient training on the importance of accurately documenting prosthetic details. Additionally, the documentation of postoperative care instructions declined from 78% to 70%. This decline can be explained by a prevalent practice in Sudan where postoperative care instructions are often documented on a separate sheet. This creates a deeply ingrained mindset among doctors to consistently document these instructions outside the main operative note. Despite the training sessions provided to address this, the habit persists. A similar phenomenon was observed at Dongola Teaching Hospital in Sudan, where a decline in postoperative care instruction documentation was also noted, even after implementing an intervention aimed at improving documentation practices [6]. This suggests that the challenge may be more systemic, influenced by long-standing habits in medical practice. To address this, future training will focus on reinforcing the importance of documenting postoperative care instructions within the main operative note, ensuring all surgical staff adopt a consistent approach.

Limitations

Our study had several limitations. First, the sample size, while adequate for demonstrating trends, was relatively small compared to other audits, such as the one by Zahid et al., with 390 operative notes in the initial cycle [9]. A larger sample size may have provided more robust conclusions and allowed for the detection of subtler trends. 

## Conclusions

Our study highlights the effectiveness of structured interventions, such as adopting a revised format and conducting training sessions, in improving the quality of surgical operative notes. By aligning with recommended guidelines, documentation accuracy significantly increased, enhancing the clarity and completeness of surgical records. However, certain areas, like the documentation of prostheses, remain unchanged, suggesting the need for continued focus on specific aspects of operative note-taking. Overall, our study underscores the importance of continuous audits and targeted improvements to maintain high standards in surgical documentation practices.
